# Preliminary evidence of olfactory signals of women’s fertility increasing social avoidance behavior towards women in pair-bonded men

**DOI:** 10.1038/s41598-017-11356-0

**Published:** 2017-09-08

**Authors:** Chen Oren, Simone G. Shamay-Tsoory

**Affiliations:** Department of Psychology, University of Haifa, 199 Aba Khoushy Ave., Mount Carmel, Haifa 3498838 Israel

## Abstract

Previous studies suggest that women’s body odor is perceived as more attractive during ovulation and that exposure to women’s chemical signals of high fertility leads to increased mating motivation. Given that pair-bonded men react differently than single men to unfamiliar women, we investigated whether women’s chemical signals of fertility influence approach behavior among pair-bonded and single men. In the first experiment, men performed the Comfortable Interpersonal Distance task while exposed to body odor samples from women who were ovulating and from the same women during their luteal phase. We found that in the presence of the body odor from ovulation, pair-bonded, but not single men, maintained greater distance from different protagonists, particularly from women. In a second experiment we exposed men to women’s body odors while they rated the attractiveness and beauty of women’s faces. Although the ratings of women’s beauty did not differ across odor conditions, when the pair-bonded men were exposed to the high fertility odor they rated highly attractive women as less sexually attractive. The results suggest that exposure to fertility cues from unfamiliar women may trigger social avoidance in pair-bonded men, an outcome that may result from identifying such cues as threats to their relationship.

## Introduction

Among various species in the animal kingdom, chemical signals are used to communicate the female’s reproductive status. In numerous mammals, female body odor as well as other body secretions become more attractive around the estrus period^1^. In this way females advertise the most fertile phase in their menstrual cycle to males^2^. In rodents, female body odors stimulate the male reproductive system by increasing male testosterone levels^3^. Similarly, in primates, exposure to females’ pre-ovulatory scents increases sexual arousal and behavior among males^4^.

Early theories suggested that in humans, women have developed an adaptation of “concealed ovulation” so that men are unable to detect their reproductive status^5^. Notwithstanding, more recent evidence reveals changes across the menstrual cycle in women’s mating preferences^6^, voice^7^, appearance^8^ and body odor^9–13^ that might serve as signals of women’s fertility. Kuukasjärv *et al*.^12^ collected body odor using cotton T-shirts from normally cycling women at different stages of their menstrual cycle. Men who further rated the T-shirts in terms of their attractiveness reported a preference for the odors collected during the days around ovulation. This effect was further attributed to axillary odors by confirming that men rated body odor collected by cotton pads placed at women’s armpits during the days around ovulation as more pleasant and attractive and less intense than odor collected during the late follicular phase of the menstrual cycle^13^. Subsequent studies have demonstrated that chemical signals of women’s fertility lead to an elevated testosterone reaction in men^14^, a reaction that is known to reflect sexual arousal^15^. In line with this, Miller and Maner^16^ showed that exposure to chemical signals of women’s fertility leads men to exhibit higher implicit accessibility to mating-related concepts and to risky decision-making in a gambling task. The authors suggested that exposure to women’s chemical signals of high fertility triggers an increased mating motivation^16^, which in turn influences men’s behavior in various ways.

A prominent feature of mating motivation is approach behavior, that is, the initiation of behavior towards positive, rewarding stimuli^17^. In a social context, approach motivation may be reflected by personal space preference. All living organisms monitor and regulate the space around them, thus constantly defining their social dynamics and interactions with others^18^. We allow people with whom we have closer relationships to maintain a closer distance to us^19^, while violations of our personal space may cause us to feel uncomfortable^20^.

Approach behavior may reflect a display of romantic interest^21^. Thus, such behavior, and particularly the preferred inter-personal space, may be influenced by mating motivation, which in turn can be regulated by signals of women’s fertility. In a recent study, Tan and Goldman^22^ demonstrated that exposure to women’s body odor from the days of ovulation increased men’s preference to sit in closer proximity to where they believed a woman would sit, a preference the researchers interpreted as a stronger tendency to approach other women.

Previous research has demonstrated that men who are in monogamous relationships react differently than single men to unfamiliar women, indicating that pair-bonded men may react differently to the odors of unfamiliar women. Pair-bonded men tend to rate other women as less attractive than do single men^23–26^. This tendency is called the *derogation effect* ^27^ since it was found to result from reduced ratings by pair-bonded men rather than from enhanced ratings by single men^25^. In line with this, following intranasal oxytocin administration pair-bonded men were found to react with avoidant behavior towards attractive women (i.e., they maintained a greater distance between themselves and attractive women)^21^. Finally, Miller and Maner^26^ revealed that while rating the attractiveness of a naturally cycling woman, single and pair-bonded men react differently to subtle cues of fertility (e.g., body odor and skin tone changes). The authors found a trend among single men, who rated women’s attractiveness as higher around the time of ovulation, though this trend was not significant. In contrast, the pair-bonded men exhibited a significant reduction in their attractiveness ratings of women around the time of their ovulation.

Given that pair-bonded men react differently than single men to unfamiliar women, in this study we investigated whether chemical signals associated with women’s fertility influence social approach among single and pair-bonded men. We conducted two experiments in which we exposed men to women’s body odor collected during two different phases of their menstrual cycle: during ovulation and during the luteal phase. In the first experiment, we exposed men to the women’s odor samples while they performed the Comfortable Interpersonal Distance (CID) task, a task assessing preferred social distance from male and female figures. In the second experiment, we exposed a different sample of men to new women’s odor samples while they rated women’s faces in terms of their beauty and sexual attractiveness. We hypothesized that exposure to women’s body odor during ovulation would lead to an increase in social approach behavior among single men and to a reduction in social approach behavior among pair-bonded men.

**Figure 1:**
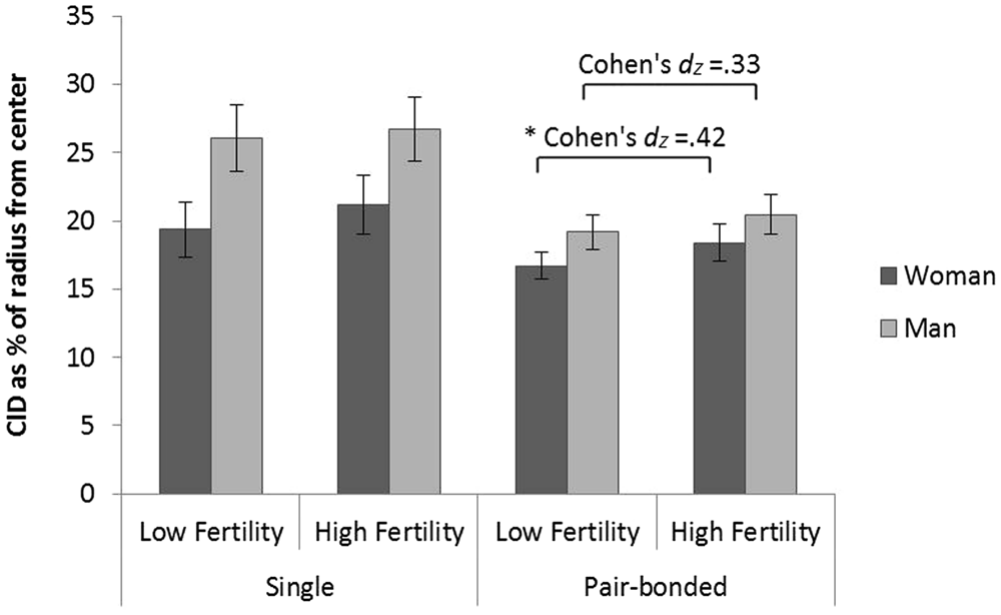
Comparison of the mean (SEM) chosen CID for male and female protagonists with respect to odor condition and relationship status. A significant difference in preferred distance was found between the high and low odor conditions. Post hoc analysis demonstrated that this effect can be attributed to pair-bonded men, who maintained a greater distance when exposed to the high fertility odor condition, especially from female protagonists. *P < 0.05, N = 65.

**Figure 2:**
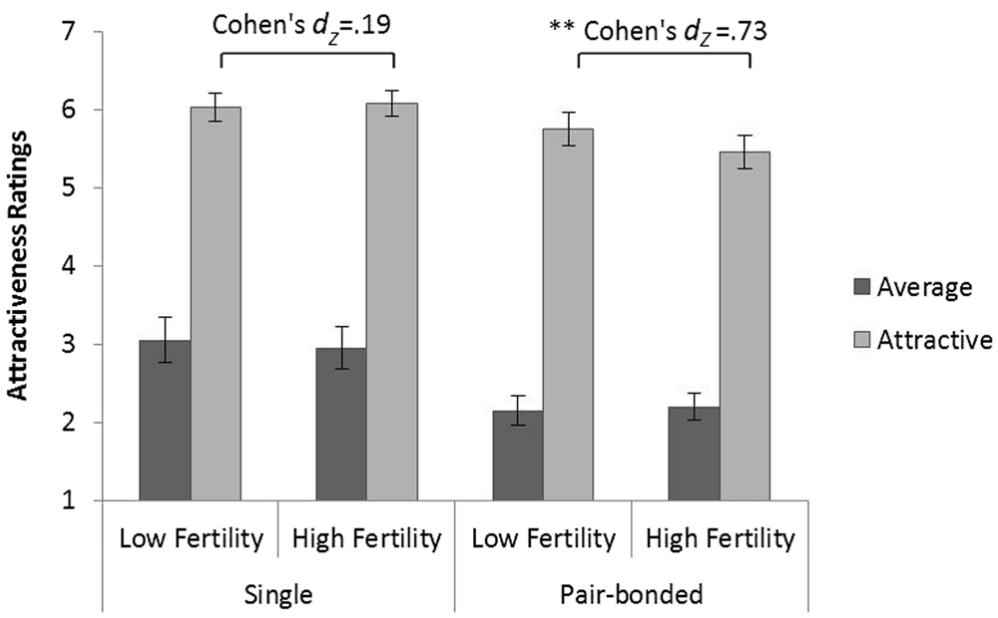
Mean (SEM) sexual attractiveness ratings for average and attractive women with respect to odor condition and relationship status. A significant difference was found between the ratings of single and of pair-bonded men, as well as a three-way interaction between odor condition, relationship status and figure’s degree of attractiveness. Pair-bonded men lower their ratings of attractive women when exposed to the high fertility odor condition. Single men rated attractive women as more sexually attractive under the high fertility odor condition, though this trend did not reach significance. **P < 0.01, N = 43.

## Results

### Experiment 1

We conducted three-way repeated-measures ANOVA, with odor condition (low and high fertility) and gender of the figure in the task as within-subject factors and relationship status as a between-subject factor. The dependent variable was the remaining distance between the figures - the distance at which the participants indicated that they began feeling uncomfortable.

We found a main effect for odor condition [F_(1,63)_ = 5.83, p = 0.019, ${\eta }_{p}^{2}$ = 0.085], with participants maintaining a greater distance between the figures under the high fertility body odor condition (M = 21.68, SD = 10.49) than under the low fertility body odor condition (M = 20.321, SD = 9.82). A main effect for figure gender was found as well [F_(1,63)_ = 26.82, p = 0.000, ${\eta }_{p}^{2}$ = 0.299], with greater distance maintained between the participant’s figure and a male figure (M = 23.09, SD = 10.80) than between the participant’s figure and a female figure (M = 18.91, SD = 9.12). The main effect for relationship status was significant [F_(1,63)_ = 4.43, p = 0.039, ${\eta }_{p}^{2}$ = 0.066], with greater distance maintained between the figures among single men (M = 23.32, SD = 12.03) than among pair-bonded men (M = 18.68, SD = 7.97). The interaction between gender of the figure in the task and relationship status was found to be significant [F_(1,63)_ = 5.64, p = 0.021, ${\eta }_{p}^{2}$ = 0.082]. The interaction between odor condition and relationship status was not significant [F_(1,63)_ = 0.051, p = 0.822, ${\eta }_{p}^{2}$ = 0.001]. The interaction between odor condition and gender of the figure in the task [F_(1,63)_ = 0.910, p = 0.344, ${\eta }_{p}^{2}$ = 0.014] and the three-way interaction between odor condition, gender of the figure and relationship status [F_(1,63)_ = 0.242, p = 0.624, ${\eta }_{p}^{2}$ = 0.004] were also not significant.

Follow-up analysis using two-way repeated-measures ANOVA demonstrated a significant main effect for odor condition among pair-bonded men [F_(1,36)_ = 6.854, p = 0.013, ${\eta }_{p}^{2}$ = 0.16], with greater distance maintained between the figures under the high fertility body odor condition (M = 19.43, SD = 8.74) than under the low fertility body odor condition (M = 17.94, SD = 7.09). The main effect for the figure’s gender was significant as well [F_(1,36)_ = 6.74, p = 0.014, ${\eta }_{p}^{2}$ = 0.16], with participants maintaining greater distance from men figures (M = 19.82, SD = 8.45) than from women figures (M = 17.55, SD = 7.35). The interaction effect between odor condition and figure gender was not significant [F_(1,36)_ = 0.33, p = 0.569, ${\eta }_{p}^{2}$ = 0.009].

A simple effects analysis using paired-sampled *t* - tests, with Bonferroni adjusted alpha of 0.025 per test, revealed a significant simple effect for odor condition when the presented figure is female [t_(36)_ = 2.505, p = 0.017, 95%CI(−3.029, −0.319), Cohen’s *d*_*Z*_ = 0.42]. Simple effect for odor condition among pair-bonded men when the presented figure is male was not significant [t_(36)_ = 2.03, p = 0.050, 95%CI(−2.599, 0.0001), Cohen’s *d*_*Z*_ = 0.33]. In both cases, the distance maintained was greater during exposure to the high fertility body odor (M = 18.39, SD = 8.37 and M = 20.46, SD = 9.09, respectively) than during exposure to the low fertility body odor (M = 16.71, SD = 6.16 and M = 19.16, SD = 7.81, respectively).

The main effect for odor condition among single men was not significant [F_(1,27)_ = 1.36, p = 0.253, ${\eta }_{p}^{2}$ = 0.048], nor was the interaction effect [F_(1,27)_ = 0.501, p = 0.485, ${\eta }_{p}^{2}$ = 0.018]. The main effect for gender of the figure was significant [F_(1,27)_ = 17.51, p = 0.000, ${\eta }_{p}^{2}$ = 0.39], with participants maintaining a greater distance for men figures (M = 26.37, SD = 12.41) than for women figures (M = 20.27, SD = 10.91). Bonferroni - corrected simple - effects were not significant for both men [t_(27)_ = 0.439, p = 0.664, 95%CI(−3.66, 2.37), Cohen’s *d*_*Z*_ = 0.08] and women figures [t_(27)_ = 1.51, p = 0.142, 95%CI(4.29, −0.648), Cohen’s *d*_*Z*_ = 0.28] (Fig. 1).

### Experiment 2

#### Women’s sexual attractiveness

Three-way repeated-measures ANOVA was conducted, with odor condition (low and high fertility) and degree of woman’s attractiveness (attractive and average looking women) as within-subject factors. Relationship status served as a between-subject factor.

The main effect of odor condition was not significant [F_(1,40)_ = 1.617, p = 0.211, ${\eta }_{p}^{2}$ = 0.039]. The main effect of degree of attractiveness was significant [F_(1,40)_ = 292.65, p = 0.000, ${\eta }_{p}^{2}$ = 0.88], with higher attractiveness ratings given to the attractive women (M = 5.838, SD = 0.896) than to the average looking women (M = 2.609, SD = 1.144). The main effect of relationship status was significant [F_(1,40)_ = 8.174, p = 0.007, ${\eta }_{p}^{2}$ = 0.170], with single men giving higher attractiveness ratings (M = 4.54, SD = 1.86) than pair-bonded men (M = 3.89, SD = 1.92). The interaction between odor condition and relationship status was not significant [F_(1,40)_ = 0.775, p = 0.384, ${\eta }_{p}^{2}$ = 0.019]. The interaction between relationship status and woman’s attractiveness [F_(1,40)_ = 0.982, p = 0.328, ${\eta }_{p}^{2}$ = 0.024] and the interaction between odor condition and woman’s attractiveness [F_(1,40)_ = 0.782, p = 0.382, ${\eta }_{p}^{2}$ = 0.019] were both not significant. The three-way interaction between odor condition, relationship status and degree of attractiveness was found to be significant [F_(1,40)_ = 5.213, p = 0.028, ${\eta }_{p}^{2}$ = 0.115].

Post-hoc analysis using two-way repeated-measures ANOVA demonstrated a significant interaction effect between odor condition and degree of women’s attractiveness among the pair-bonded men [F_(1,19)_ = 8.833, p = 0.008, ${\eta }_{p}^{2}$ = 0.317], as well as a main effect for degree of attractiveness [F_(1,19)_ = 145.877, p = 0.000, ${\eta }_{p}^{2}$ = 0.885], with higher attractiveness ratings assigned to attractive women (M = 5.6, SD = 0.938) than to average looking women (M = 2.175, SD = 0.786). The main effect for odor condition was not significant [F_(1,19)_ = 2.497, p = 0.131, ${\eta }_{p}^{2}$ = 0.116]. Bonferroni-corrected simple-effect analysis using paired-sampled *t-*tests revealed a significant effect for odor condition in ratings of attractive women [t_(19)_ = 3.21, p = 0.005, 95%CI(−0.104, 0.496), Cohen’s *d*_*Z*_ = 0.73], with lower rating given to these women during exposure to the high fertility body odor condition (M = 5.45, SD = 0.944) than during exposure to the low fertility body odor condition (M = 5.75, SD = 0.931). The simple effect of odor condition in ratings of the average looking women was not significant [t_(19)_ = −0.483, p = 0.635, 95%CI(−0.267, 0.167), Cohen’s *d*_*Z*_ = 0.01].

Among single men, the main effect of odor condition was not significant [F_(1,21)_ = 0.073, p = 0.790, ${\eta }_{p}^{2}$ = 0.003], nor was the interaction effect between odor condition and degree of attractiveness [F_(1,21)_ = 0.726, p = 0.404, ${\eta }_{p}^{2}$ = 0.033]. The main effect for degree of attractiveness was significant [F_(1,21)_ = 145.81, p = 0.000, ${\eta }_{p}^{2}$ = 0.874], with single men giving higher attractiveness ratings to attractive women (M = 6.055, SD = 0.806) than to average looking women (M = 3.005, SD = 1.276). According to Bonferroni-corrected simple-effects analysis, odor condition demonstrated a non-significant trend in ratings of attractive women [t_(21)_ = −0.880, p = 0.389, 95%CI(−0.183, 0.074), Cohen’s *d*_*Z*_ = 0.19], with higher attractiveness ratings during exposure to the high fertility body odor condition (M = 6.082, SD = 0.772) than during exposure to the low fertility body odor condition (M = 6.027, SD = 0.856). The simple effect of odor condition in ratings of average-looking women was not significant [t_(21)_ = 0.611, p = 0.548, 95%CI(−0.240, 0.440), Cohen’s *d*_*Z*_ = 0.13] (Fig. 2).

#### Women’s beauty

Three-way repeated-measures ANOVA was conducted, with odor condition (low and high fertility) and degree of attractiveness (attractive and average looking women) as within-subject factors. Relationship status served as a between-subject factor.

**Figure 3:**
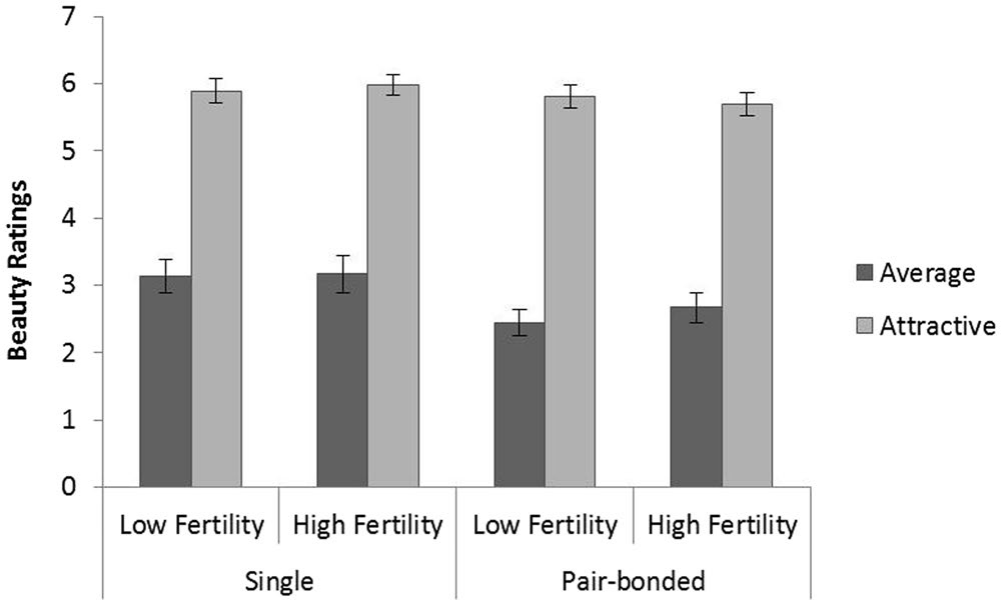
Mean (SEM) beauty ratings for average and attractive women with respect to odor condition and relationship status. No significant difference was found. N = 43.

The main effect of odor condition was not significant [F_(1,40)_ = 0.403, p = 0.529, ${\eta }_{p}^{2}$ = 0.01]. Neither were the main effect for relationship status [F_(1,40)_ = 3.33, p = 0.075, ${\eta }_{p}^{2}$ = 0.077] and the interaction between odor condition and relationship status [F_(1,40)_ = 0.000, p = 0.998, ${\eta }_{p}^{2}$ = 0.000]. A significant main effect was found for degree of attractiveness of the presented woman [F_(1,40)_ = 335.469, p = 0.000, ${\eta }_{p}^{2}$ = 0.893], with higher ratings given to attractive women (M = 5.843, SD = 1.169) than to average looking women (M = 2.855, SD = 0.761). The interaction between odor condition and attractiveness, as well as the three-way interaction, were not significant [F_(1,40)_ = 0.768, p = 0.386, ${\eta }_{p}^{2}$ = 0.019; F_(1,40)_ = 1.497, p = 0.228, ${\eta }_{p}^{2}$ = 0.036, respectively] (Fig. 3).

## Discussion

In this study we investigated the effect of women’s chemical signals of fertility on approach behavior, in particular on preferred inter-personal distance and on beauty and sexual attractiveness ratings of unfamiliar women. We focused particularly on the role of relationship status on moderating these effects. Due to the large variations in the menstrual cycle across different women, in our study we used hormonal tests to confirm the occurrence of ovulation. We used a relatively large sample of women donors and mixed odor stimuli in order to avoid individual preferences between donors and recipients^28^. In line with our hypothesis, we found that exposure to women’s chemical signals of high fertility increased the inter-personal distance preferred by pair-bonded men, especially the distance they maintained from female protagonists. In the second experiment, we found that exposure to women’s chemical signals of high fertility led pair-bonded men to assign lower sexual attractiveness ratings to highly attractive women but had no effect on their ratings of women’s beauty. It is interesting to note that we found no significant effects in the single men groups. As hypothesized, we did find a non-significant trend showing that single men had higher attractiveness ratings of attractive women during the high fertility body odor condition than during the low fertility body odor condition. This effect may not have been detected statistically due to a ceiling effect in single men’s rating of attractive women.

The main effect found for the gender of the figure in the CID task indicates that participants successfully discriminated between the male and female figures. As expected, participants maintained greater distance between themselves and male figures as compared to female figures. This is in line with previous findings demonstrating that opposite-sex pairs require less interpersonal distance than male-male pairs^29, 30^, possibly due to women’s tolerance for violations of their personal space^31^ or to differences in the perceived meaning of physical closeness between same-sex and different-sex pairs^32^.

The results observed among the pair-bonded men may be explained by a strong motivation to maintain their current relationship. As a major potential threat to the stability of relationships is attractive alternatives of the opposite sex^23^, it is possible that the men perceived exposure to odors of other women’s fertility as a threat to their current relationships, compelling them to react with avoidant behavior toward the threat. This mechanism is similar to that of the derogation effect discussed previously^27^, in which pair-bonded men tend to down-regulate arousal caused by threats to their relationship.

Previous research has claimed that women’s chemical signals of high fertility increase mating motivation among exposed men^16^. Elaborating on this model, we suggest that the heightened mating motivation influences approach behavior. We further propose that the effects of women’s chemical signals of high fertility on approach behavior is moderated by the relationship status of the exposed men, which influences how they cope with the increased mating motivation, thereby creating different behavioral outcomes.

A possible mechanism for this difference may involve changes in testosterone levels. Previous studies suggest that testosterone levels are related to mating efforts^33^ and to sexual arousal in men^15^ and women^34^. Other research findings show that basal testosterone levels are lower among men who are in a committed relationship than among single men^35, 36^. Recent studies demonstrate that exposure to women’s body odor from the time of ovulation causes elevation in testosterone levels in receiver men^14, 37^. This finding supports the notion that exposure to women’s body odor enhances sexual arousal and mating motivation. It is possible that pair-bonded men are usually able to down-regulate the effects of increased mating motivation^38^, thus avoiding its behavioral expression. This is in line with Tan *et al*.^22^, who found higher approach behavior following exposure to women’s chemical signals of high fertility when the men believed they were intoxicated, an effect that might also occur in other settings with low resources for self-regulation^24^. In addition, men’s testosterone levels correlate positively with sexual arousal thresholds and sexual interest^39–41^. It is possible that with higher basal levels of testosterone, single men have lower thresholds for sexual arousal, thus reacting to both types of women’s odor stimuli to the same extent.

Our results indicate that the effect of chemical signals of women’s fertility on the approach behavior of pair-bonded men could be attributed in part to an increase in the preferred inter-personal distance from male protagonists. This finding could be interpreted as an increase in behaviors that promote maintenance and preservation of one’s territory. It is well established that in territorial mammalian species, males make more scent marks in a territory when they are exposed to odorous cues of estrus females^42, 43^. Such marking behavior has been found to play a role in the establishment of territories^42^. Thus, it is possible that the behavior of the men in our experiment in maintaining more distance from male protagonists reflects an increase in territorial behavior as a result of exposure to female high fertility cues.

In line with previous studies^23–26^, in our sample we found a significant effect of relationship status on women’s attractiveness ratings. Women’s beauty ratings did not differ significantly across the relationship status groups. Interestingly, the ratings of women’s beauty did not differ between the odor conditions, while women’s sexual attractiveness ratings did differ across the odor conditions in the group of pair-bonded men. According to Ferdenzi *et al*.^44^, the terms “beauty” and “attractiveness” are often used as equivalent though they have different meanings. While “beauty” refers to aesthetic pleasure triggered by the features of the presented object (such as the body or the face), the term “sexual attractiveness” reflects the motivation “to approach” someone^45^ and therefore includes a biological response of sexual arousal^46^. Our findings suggest that women’s body odor during periods of high fertility has a greater impact on the arousal component of the assessed women rather than the aesthetic components, thereby influencing the participant’s motivation to approach other women.

Another possible underlying mechanism is the oxytocinergic system. In a similar experiment, Scheele *et al*.^21^ demonstrated that intranasal administration of oxytocin stimulates pair-bonded men to maintain a greater distance from an attractive woman. It is possible that exposure to women’s body odor during high fertility leads to an elevated oxytocin reaction in the receiver, thus selectively influencing expression of approach motivation in the group of pair-bonded men. Further studies addressing this new hypothesis are warranted.

It should be noted that the current findings contradict the findings of Tan *et al*.^22^ who reported increased approach behavior among men following exposure to women’s odors during periods of high fertility. Given that the paradigm used by Tan and Goldman examined active approach toward women, no threat signaling was involved. The task used in our study may have induced a more threatening situation in which the participant is being approached by unfamiliar figures who are intruding on his personal space, thus mainly activating the avoidance system and resulting in a greater distance being maintained between the figures.

In conclusion, our findings show that women’s body odor from high fertility periods differentially influences approach behavior among pair-bonded and single men. The high fertility odor mainly influences men who are currently in a relationship, encouraging them to adopt an avoidant attitude toward social stimuli, especially stimuli that involve other women. These findings advance our understanding of the action of chemical signals in the social environment.

## Materials and Methods

### Experiment 1

#### Participants

A priori sample size estimates indicated for a power of 0.80, a minimum of 34 participants at each group to detect a medium effects size (Cohen’s *d*_*Z*_ = 0.50), therefore we aimed to recruited 68 participants. To this end, sixty-seven men were recruited via advertisements posted at the University of Haifa and in the social media. The participants completed online screening questionnaires aimed to determine their suitability for the experiment.

Male participants ranged from 19 to 35 years old (M = 26.37, SD = 3.28), and were native Hebrew speakers. They reported having normal or corrected to normal vision and a normal sense of smell. They further reported that they were heterosexual, non-smokers and healthy and that they had not been sick during the last three weeks.

The study was approved by the University of Haifa Ethics Committee. All the experimental procedures described below were conducted in accordance with institutional ethical guidelines. Participants signed an informed consent form and were given payment or academic credit for their time.

#### Odor collection

Forty-three female participants ranging in age from 19 to 31 years old (M = 24.73, SD = 2.33) were recruited for collection of a pool of body odor samples. The women were recruited after having confirmed that they do not use hormonal contraceptives and that they have a regular menstrual cycle. All women reported that they were heterosexual, were not currently living with a spouse, were non-smokers and do not use medications or suffer from any physical or a mental disease.

Unlike previous studies in which the timing of ovulation was speculated by counting the menstrual cycle days^13, 14, 16, 47^, in this study we used hormonal ovulation examination in order to confirm the occurrence of ovulation. Woman used a commercial urine ovulation test (ZER Hitech LTD) to trace their menstrual cycle. They collected their body odor using cotton T-shirts^12, 14, 16^ at two phases of the menstrual cycle: at ovulation and at the luteal phase. Women were requested to begin using the ovulation test on the 11^th^ day from the beginning of menses. On the night when the ovulation test was positive, indicating that process of ovulation had begun, women were asked to shower thoroughly without using soap or shampoo and to refrain from using deodorant, perfumes or lotions following that shower. They were further asked to refrain from smoking, drinking alcohol, eating odorous food (such as garlic, onion, asparagus and odorous spices) and engaging in activities that might produce odors (such as cooking or sexual intercourse). These instructions were similar to those given in previous studies^9, 11–14, 16, 48–50^. Following the shower, the women put on a new, odorless, cotton T-shirt that had previously been kept in a sealed plastic bag. The women were asked to put the T-shirt back in the bag in the morning and to seal it tightly. The armpit areas were cut from each T-shirt and were further cut into 12 equal pieces. So that all the male participants would receive the identical stimulus, the T-shirt pieces from the first 23 women served as a pooled stimulus for the high fertility body odor condition. The T-shirt pieces from the following 20 women were mixed into a separate pooled stimulus for the same odor condition. One week after the positive test (during the luteal phase of the menstrual cycle) the women repeated the procedure. Pools of these T-shirts served as pooled stimuli for the low fertility body odor condition^51^. All stimuli were kept in a freezer when not in use (at approximately −20 °C).

#### Odor presentation

The odor was presented using an original device. The odor stimuli were placed in a glass jar covered with non-transparent paper. The jar was sealed by a cover that was connected to both an air pump (AS-1061, Chuang Xing Manufacturing) and a nasal cannula (Over-the-Ear nasal cannula with 7 ft star lumen, Hudson RCI®). The pump continually compressed fresh air through the jar into the participants’ nostrils throughout the session.

#### Experimental task

We used a computerized version of a task assessing comfortable inter-personal distance, based on a previously validated paper-and-pencil task^52, 53^. The task was programmed using the E-prime 2.0 software package^54^. In the original task participants were presented with a circle and asked to imagine themselves standing at its center. They were asked to mark where they would want another figure to stop as it approached the center of the circle along a radius at eight different angles (0°, 45°, 90°, 135°, 180°, 225°, 270° and 315°). In the computerized task^55^, the participants were shown a fixation cross for 500 ms, followed by a circle diagram representing a room, with line-figure protagonists at the center and at one of eight entrances along the perimeter of the circular room. The participant was asked to imagine himself as the protagonist in the center of the room and to use the computer mouse to mark where he would start feeling uncomfortable as another protagonist approached him. This task was recently validated in our lab and exhibited high correlations between performance on the computerized task and performance on a behavioral task involving approaching confederates in an actual room^56^.

**Figure 4:**
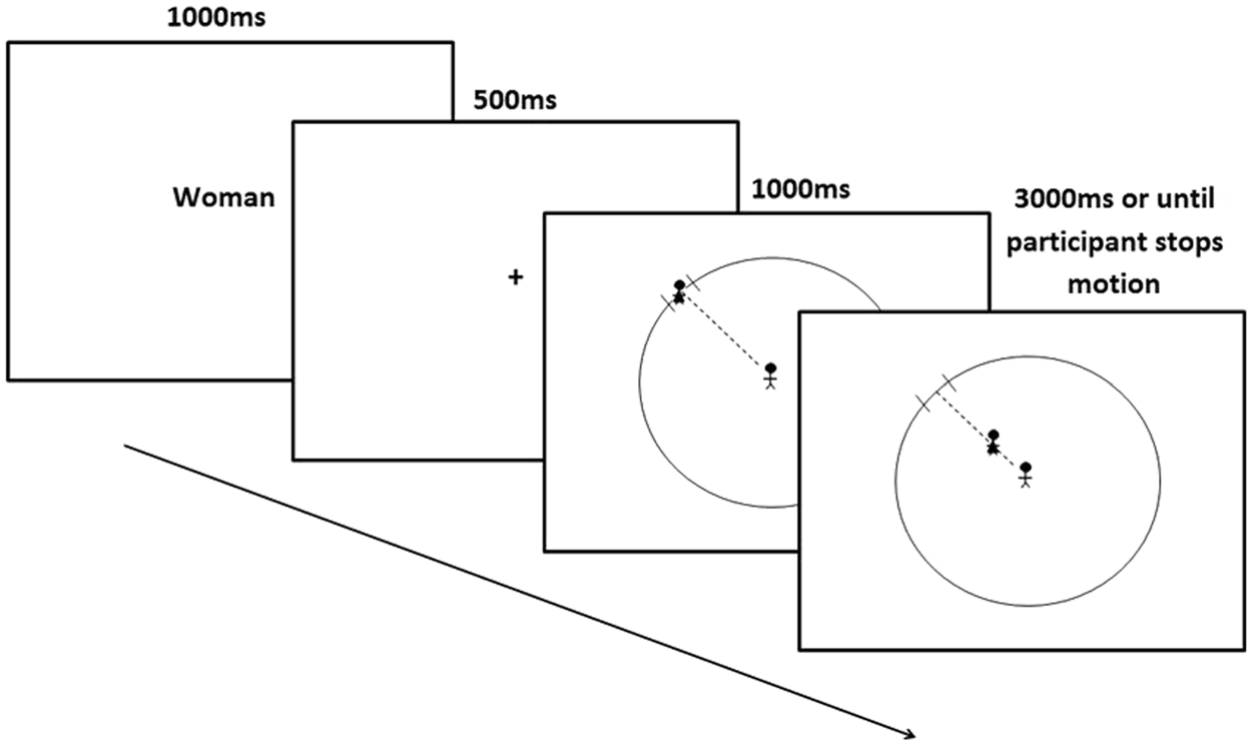
A trial from the CID task. Participants were shown the protagonist that would be approaching them during the current trial. Then, a fixation cross was presented, followed by a diagram of a circular room. Participants were asked to imagine themselves as the figure standing at the center of the room while another protagonist approaches them from the perimeter toward the center. They were requested to press a key when the protagonist reached the place they would begin to feel uncomfortable.

In this study we further defined the protagonists entering the room to be either a man or a woman. At the beginning of each trial, the participant was shown the protagonist that would be present in the circular room on the successive trial (a man or a woman), for 1000 ms. During the next 500 ms the participant was presented with a fixation cross, which was followed by a 1000 ms still presentation of the room and a 3000 ms animation of a protagonist approaching the center of the room. Female protagonists were represented with the common stick-figure wearing a dress whereas males were represented by a stick figure not wearing a dress (Fig. 4). The radius of the room was 45 mm, and the length of the line figures (both the man and the woman) was 6 mm. Following four practice trials, a total of 32 trials were presented, with each approaching protagonist appearing 16 times (two repetitions for each of the eight radii angles). The remaining distance between the protagonists was calculated by the place the participant stopped the approaching figure and was represented as the percentage of the total radius. Trials in which the remaining distance was either 0% or 100% of the total radius were removed. An average of the 16 trials for each protagonist was calculated for each participant. In order to detect potential outliers, scores of the remaining distance were standardized. Two participants with scores that were three or more standard deviations from the average were removed from the analysis.

#### Experimental procedure

We used a double-blind, within-subject design. Each participant underwent two experimental sessions within a 1-week interval. A female experimenter who was using hormonal contraceptives at the time of the experiment in order to avoid changes resulting from her menstrual cycle^12, 47^ introduced the participants to the experimental procedure. The first 36 male participants were exposed to body odors from the first 23 women, while the next 29 were exposed to odors from the next 20 women. During the first session, while the men performed the CID task they were exposed to one body odor stimulus, either from the high fertility condition or from the low fertility condition. During the second session, the participants followed a similar procedure while exposed to the other body odor condition. That is, if the stimulus in the first session was from the high fertility condition, the participant was exposed to the low fertility stimulus in the second session and vice versa. The order of odor condition presentation was counterbalanced.

### Experiment 2

#### Participants

With no available estimation for the effect size, the number of participants was based on a previous study investigating how subtle cues of fertility affect attractiveness rating^26^. Forty-two male and 20 female participants were recruited via advertisements posted at the University of Haifa and in the social media. Female participants ranged in age from 22 to 31 years (M = 24.89, SD = 2.56) and male participants ranged in age from 19 to 35 years (M = 26.17, SD = 3.63). The participants completed online screening questionnaires aimed to determine their suitability for the experiment. Their reports met the same criteria as in the first experiment. The study was approved by the University of Haifa Ethics Committee. All the experimental procedures described below were conducted in accordance with institutional ethical guidelines. The participants signed an informed consent form and were given payment or academic credit for their time.

#### Experimental task

We used a computerized version of a face-rating task based on the task in Aharon *et al*.^57^. The version used in the current experiment included 20 pictures of women’s faces. Half the pictures were of women who had been previously rated as beautiful and half had been rated as average^57^. The pictures were shown for 750 ms, in randomized order. Following presentation of each picture, the participants were asked to rate the degree to which they found each woman to be beautiful and sexually attractive, on a scale ranging from “1” (not at all) to “7” (very much). For each question, the participants’ ratings were averaged separately for the beautiful and the average-looking women.

#### Experimental procedure

The body odor collection procedure was the same as described in Experiment 1. We used a double-blind, within-subject design. The participants underwent two experimental sessions within a 1-week interval. In each session they were exposed to one of the two odor conditions. Odor exposure was the same as in Experiment 1. During each session, the participants performed the picture-rating task. The order of odor condition presentation was counterbalanced.

### Data Availability

The datasets generated and analyzed during the current study are available from the corresponding author on reasonable request.
